# Optimisation of Low-Toxicity Solvent Employment for Total Lipid and Tocopherol Compound Extraction from Patagonian Squid By-Products

**DOI:** 10.3390/foods12030504

**Published:** 2023-01-21

**Authors:** Alicia Rodríguez, Marcos Trigo, Santiago P. Aubourg, Isabel Medina

**Affiliations:** 1 Department of Food Science and Chemical Technology, Faculty of Chemical and Pharmaceutical Sciences, University of Chile, C/Santos Dumont 964, Santiago 8380000, Chile; 2 Department of Food Technology, Marine Research Institute (CSIC), c/E. Cabello 6., 36208 Vigo, Spain

**Keywords:** by-products, *Doryteuthis gahi*, total lipids, tocopherols, extraction, ethanol, acetone, ethyl acetate, optimisation, simplex-lattice design

## Abstract

The extraction of total lipids and tocopherol compounds from Patagonian squid (*Doriteuthis gahi*) by-products (viscera, heads, skin, etc.), resulting from squid mantel commercialisation, was studied. An optimisation simplex-lattice design by employing low-toxicity solvents (ethanol, acetone, and ethyl acetate) was carried out taking into account their relative concentrations. The variance analysis of data showed that the quadratic model was statistically significant (*p* < 0.05); empirical coded equations were obtained as a function of the low-toxicity solvent ratios. The optimised lipid extraction was obtained by employing the 0.642/0.318/0.040 (ethanol/acetone/ethyl acetate) solvent ratio, respectively, leading to an 84% recovery of the total lipids extracted by the traditional procedure. In all extracting systems tested, the presence of α-, γ-, and δ-tocopherol compounds was detected, α-tocopherol being the most abundant. For α-, γ-, and δ-tocopherol compounds, the optimisation process showed that acetone extraction led to the highest concentrations in the lipid extract obtained (2736.5, 36.8, and 2.8 mg·kg^−1^ lipids, respectively). Taking into account the recovery yield on a by-product basis, the values obtained for the three tocopherols were included in the 88.0–97.7%, 80.0–95.0%, and 25–75% ranges, respectively, when compared to the traditional extraction. This study provides a novel and valuable possibility for α-tocopherol extraction from marine by-products.

## 1. Introduction

Processing marine fish and invertebrate generates a great amount of by-products, which constitutes an important source of environmental contamination [1,2]. Nowadays, the valorisation of by-products is considered to be of high interest because such biomass substrates have been shown to contain valuable components [3,4]. Thus, recent studies have reported that head, skin, viscera, tails, blood, shell, etc., include a wide range of bioactive components that may be incorporated into nutraceutical, functional food formulation, or pharmaceutical applications [5,6]. The development of advanced technologies for the extraction and purification of high added-value molecules, such as bioactive peptides, enzymes, minerals, and antioxidants, constitutes a current challenge for seafood technologists [7].

To obtain marine oils and fats, a wide range of traditional and advanced methods have been used, such as wet pressing [8,9], pH adjustment [10], supercritical fluid extraction [11], or enzymatic hydrolysis [12]. In recent decades, there has been increased interest in the development of low-toxicity methods in order to reduce the risk of chemical exposure to humans and the environment. Among them, great attention has been accorded to the use of eco-friendly solvents for lipid extraction. Remarkably, such efforts have been especially addressed to agricultural by-products and have employed solvents such as ethanol, acetone, glycerol, and ethyl acetate [13,14,15]. Concerning marine products, previous research on low-toxicity solvent employment can be considered scarce. Thus, Gigliotti et al. [16] checked acetone/ethanol mixtures for lipid fraction extraction from Antarctic krill (*Euphausia superba*). Additionally, Bian et al. [17] optimised the subcritical n-hexane/isopropanol extraction of lipids from microalga *Scenedesmus obliquus* to maximise lipid and fatty acid yield. Recently, several combinations of non-toxic solvents were tested as extracting solvent mixtures to obtain edible oils and antioxidants (i.e., vitamin E) from microalga (*Scenedesmus dimorphus*) [18].

Vitamin E is a natural and most effective lipid-soluble antioxidant that refers to a group of eight chemically related compounds (four tocopherols, α, β, γ, and δ; four tocotrienols, α, β, γ, and δ) possessing a 6-chromanol ring structure attached to a carbon side chain [19,20]. Based on their important role as lipid-soluble chain-breaking antioxidants, tocopherol compounds have received great attention from marine technologists [21]. Among them, α- tocopherol has been found to be the most abundant in marine animals, especially in vertebrate ones [22]. As a lipophilic antioxidant, it has shown an important role in vivo [23] in the stability of food fat and oils [24]. Considerable differences in α-tocopherol concentration have been reported between light and dark fish muscle, the latter presenting the highest values [25], while fatty fish species have shown greater levels than lean ones [26]. Remarkably, shellfish species are reported to provide scarce quantities. According to this protecting role of unsaturated fatty acids from oxidation, α-tocopherol content in fish muscle has been shown to decrease as a result of quality loss during processing and storage [27,28].

Cephalopod species represent a great economic resource in many countries. Among squid species, Patagonian squid (*Doriteuthis gahi*) is a neritic species that can be widely distributed in the Atlantic and Pacific coasts of South America. Recently, this species has attracted important interest as a fishery resource [29]. After excision of the mantle tissue, all other body parts are considered by-products and are commonly assigned to meal production. However, a recent seasonal study of *D. gahi* by-products showed a remarkable presence of high-value constituents, such as polyunsaturated fatty acids, α-tocopherol, phospholipids, and essential macroelements and trace elements [30]. Recently, lipid extracts including high levels of phospholipids and ω3 fatty acids were obtained from Patagonian squid by-products by employing different kinds of low-toxicity solvents [31].

The present research focused on the extraction of total lipids and tocopherol compounds from Patagonian squid by-products by employing low-toxicity solvents (ethanol, acetone, and ethyl acetate). To optimise the extraction yield, a simplex-lattice design was carried out, taking into account different solvent ratios. To the best of our knowledge, no previous research is available related to tocopherol compound extraction from marine by-products.

## 2. Materials and Methods

### 2.1. Initial Squid and Sampling Procedure

The initial squid samples were captured near the Argentinean coast at the South-West Atlantic Ocean, on-board frozen (−40 °C), and transported to the SERPESBA S. L. U. (Vigo, Spain) factory. Then, the samples were thawed (overnight storage at 4 °C), the mantel was taken for commercialisation purposes, and the resulting by-products (heads, skin, viscera, tails, etc.) were pooled together and carried in refrigerated conditions (4 °C) to our laboratory (Marine Research Institute, Vigo, Spain).

To assess the moisture and lipid values of the initial by-products, four 15 g portions of by-products were separated and subjected independently (*n* = 4) to the traditional procedures according to the methodology described in Section 2.3.

Meanwhile, two 1 kg portions of by-products were lyophilised (−70 °C, 72 h, 0.05 mTorr) (Model FD8515-C60, Ilshin Biobase Europe, Ede, The Netherlands). Then, the lyophilised samples were pooled together and utilised for lipid extraction by employing different extracting systems according to the experimental design expressed in Section 2.2.

According to a previous studies related to marine substrates [16,31,32], the lyophilisation process is considered necessary to facilitate lipid extraction by employing relatively polar solvents (i.e., acetone and ethanol) as in the present case and to, therefore, increase lipid yield.

### 2.2. Experimental Design for Lipid Extraction by Low-Toxicity Solvents

The low-toxicity solvents chosen for the current study were ethanol, acetone, and ethyl acetate. A simplex-lattice mixture design was carried out, including three experimental factors (the proportion of each of the low-toxicity solvents in the extracting mixture) and four response variables (total lipid yield and the content of the three tocopherol compounds detected, i.e., α, γ, and δ).

The different extracting conditions applied to lyophilised squid by-products are described in Table 1, where the relative solvent content in each extracting condition is indicated (0–1 range). The design consisted of a total of fourteen experiments, including six different experimental conditions (different low-toxicity solvent ratios) carried out in duplicate, and two centroids including the same concentration for each of the low-toxicity solvents employed. Each of the extracting conditions (from number 1 to number 14) was carried out in duplicate. Furthermore, extraction of the lipid fraction was also carried out by the traditional procedure (1/1 chloroform/methanol, *v*/*v*) of lyophilised by-products.

### 2.3. Moisture Determination and Traditional Lipid Extraction

The moisture content was determined in the initial by-products and in the lyophilised by-products (1–2 g) according to the official method 950.46B [33]. For this, the weight difference before and after heating (4 h at 105 °C) was determined. Results were calculated as g·kg^−1^ wet squid by-products.

The traditional lipid extraction of the wet by-products and the lyophilised by-products was carried out according to the Bligh and Dyer [34] method. For this, a single-phase solubilisation of the lipids was employed using a chloroform/methanol (1/1) mixture. Lipid extracts were quantified according to method proposed by Herbes and Allen [35]. Results were calculated as g·kg^−1^ dry by-products.

### 2.4. Lipid Extraction with Low-Toxicity Solvents

The lipid extraction of lyophilised by-products with low-toxicity systems was carried out according to the above-mentioned design (Table 1) as follows: 3.5 g of lyophilised by-products and 10 mL of the solvent system were mixed, stirred (1 min at 4 °C), and centrifuged (3500× *g* for 10 min at 4 °C), and the supernatant was collected. This procedure was repeated two more times and all supernatants were pooled together. A partial evaporation of the solvent mixture (rotary evaporator; 10 min at 30 °C) was carried out in all extracts, and all extracts were brought up to a 15 mL volume and stored at −40 °C before analysis.

In all kinds of low-toxicity lipid extracts, lipid quantification was carried out by the method proposed by Herbes and Allen [35]. Results were calculated as g·kg^−1^ dry by-products.

### 2.5. Tocopherol Analysis

The profile of tocopherol compounds in squid by-products was determined by the method proposed by Cabrini et al. [36]. For this purpose, the lipid fraction obtained from the lyophilised squid by-products by each of the extracting systems (low-toxicity and traditional) was carried out to dryness under nitrogen flux, dissolved in isopropanol, and analysed by HPLC (ODS column, 15 cm × 0.46 cm i.d.). The column was fluxed with methanol for 2 min, then a gradient from 0 to 50% of isopropanol was applied for 10 min. A 1.5 mL·min^−1^ flow rate was employed and detection was carried out at 280 nm. The possible presence of *α-*, *β-*, *γ-,* and *δ-*tocopherol molecules was checked. For quantitative purposes, the content of each tocopherol present in the extracting systems was calculated with calibration curves prepared from the corresponding commercial tocopherol compound and calculated as mg·kg^−1^ lipids and mg·kg^−1^ dry by-products.

### 2.6. Statistical Analysis

Data (*n* = 4) obtained from total lipid yields and the content of the tocopherol compounds detected were analysed by employing one-way ANOVA (*p* < 0.05) to investigate differences between the different kinds of lipid extracts tested (traditional and low-toxicity systems) (Statistica version 6.0, 2001; Statsoft Inc., Tulsa, OK, USA). A least-squares difference (LSD) method was applied to carry out the comparison of means.

A statistical analytical system was employed for multiple regression analyses, ANOVA, canonical analysis, and analysis of ridge maximum of data in the response surface regression (RSREG) procedure. Estimated response surfaces and the contours of the estimated response surfaces were developed using the fitted quadratic polynomial equations obtained from RSREG analyses [37]. The 95% confidence intervals of each parameter yield were calculated, taking into account the number of replicates and the standard deviation of each sample.

The interaction of the content ratio of the three solvents was modelled using an approach based on experiments with mixtures [38]. In this case, a {3,2} simplex-lattice mixture design was found suitable for this purpose [39], where the first number in the bracket refers to the number of solvents, and the second number refers to a second-degree model that was used to estimate the parameters of the model. In particular, the Scheffé second-degree model [40] was found to be suitable for this purpose and can be expressed according to Equation (1):

Y = β_1_X_1_ + β_2_X_2_ + β_3_X_3_ + β_1,2_X_1_X_2_+ β_1,3_X_1_X_3_ + β_2,3_X_2_X (1)

where Y = response variable; β_i_ = coefficient parameter of each single solvent (linear terms); β_i,j_ = coefficient parameter of each of the three solvent mixtures (non-linear terms); and X_i_ = proportion of the components expressed in a 0–1 range. The special cubic model was employed to add terms involving products of three components. Statgraphics 18^®^ software (Copyright 1982–1917 by Statgraphics Technologies, Inc., Rockville, MD, USA) was used [41].

## 3. Results and Discussion

### 3.1. Total Lipid Extraction

The initial squid by-products showed levels of 859.3 ± 3.94 and 15.5 ± 0.11 g·kg^−1^ for moisture and total lipids, respectively. Such values are in agreement with previous research concerning whole by-products obtained from processing the current squid species [30,31]. These moisture and lipid contents can be considered very similar to those present in edible tissues corresponding to low-fat marine species [22,42]. On the contrary, the liver tissue obtained from other squid species showed substantially higher lipid values (15–57 g·100 g^−1^ tissue) than the present by-products [43,44].

Once lyophilised, lipid extraction of squid by-products by the traditional procedure (i.e., chloroform/methanol) led to a total lipid presence of 90.6 g·kg^−1^ dry by-products (Table 2). The employment of any of the low-toxicity solvents tested provided lower (*p* < 0.05) levels of total lipids than in the case of the traditional procedure (Table 2). Notably, the highest lipid values (*p* < 0.05) were obtained in all the extracting systems, including ethanol. Thus, a combination of ethanol with acetone and/or ethyl acetate led to 76–80% of the lipid content obtained by employing the traditional extracting procedure. On the contrary, low-toxicity systems that did not include ethanol led to a lower (*p* < 0.05) recovery (33–41%); the lowest values (*p* < 0.05) were obtained in the case of employing acetone as a single extracting solvent.

A lower lipid extractability of low-toxicity systems than in the case of the traditional procedure can be explained by taking into account the fact that low-toxicity solvents tested are more polar than the chloroform/methanol system and, therefore, would not be likely to completely extract the non-polar lipid classes, such as triacylglycerols, waxes, cholesterol esters, etc. Consequently, a relative increase in the presence of polar lipid classes would be produced in the resulting lipid extract. As previously mentioned, remarkable differences were obtained among the different low-toxicity extracting systems tested. According to previous research [30], by-products from the current squid species have shown that phospholipids are the most abundant lipid group (359–464 g·kg^−1^ lipids), followed by other lipid classes that can be considered relatively polar (i.e., free fatty acids and sterols, 157–282 and 115–132 g·kg^−1^ lipids, respectively). Based on the fact that ethanol is the most polar solvent of all low-toxicity solvents tested in the current study, ethanol-containing systems could lead to a higher extraction of such polar lipid compounds; therefore, they could lead to a higher lipid yield than in the case of solvent mixtures that do not include this solvent.

Similarly, Gigliotti et al. [16] checked the lipid fraction extraction of a polar-rich lipid substrate (Antarctic krill, *Euphausia superba*) with different acetone/ethanol ratios. As in the present study, an increasing presence of ethanol in the extracting system led to a lipid yield increase so that higher lipid levels (g·100 g^−1^ freeze-dried krill) were obtained by employing solvent ratios (*v*/*v*) of 1/30 (ca. 13.) and 1/12 (ca. 12.0) than with 1/9 (ca. 9.5) and 1/6 (ca. 7.5) ratios. Furthermore, Jiménez Callejón et al. [32] focused on the extraction of polar-rich lipid fractions from microalgal biomass by carrying out a two-step extraction. In the first one, hexane was employed to remove neutral lipids from the microalgal substrate, while the second step, consisting of ethanol extraction of the resulting pellet, led to a lipid extract with a high concentration of polar lipids (i.e., phospholipids and glycolipids). Lipid extraction with hexane/isopropanol (3:2, *v*/*v*) from microalga *S. obliquus* was studied by applying subcritical conditions [17]. The optimised lipid yield was reached at 85 °C and 1.5 MPa conditions, leading to an 82.6% recovery when compared to the traditional procedure. Recently, Li et al. [18] carried out a comparative lipid extraction from microalga *S. dimorphus* by employing several extracting systems (ethanol/hexane, 3:2; ethyl acetate/hexane, 1:1; methanol/hexane, 1:0.8; hexane; aq. 95% ethanol). According to the current research, the highest lipid yields were obtained by applying 95% ethanol, which led to an 80% recovery when compared to the traditional procedure.

Based on the simplex-lattice design developed in the present study, the variance analysis of data corresponding to the lipid content was carried out. As a result, the quadratic model showed to be statistically significant (*p* < 0.05) (Table 3). The variance analysis also indicated that the fitted model explained 99.25% of the variability of the lipid value (R-squared value), with the adjusted R-squared value being 98.79%. Furthermore, the standard error of the estimate (SEE) showed that the standard deviation of the residuals was 2.0072, which approximately indicates the prediction errors (residuals) that can be considered in the present data set. Because this SEE value was low, it indicated that forecasts and predictions were accurate in the current study for total lipid content.

**Figure 1:**
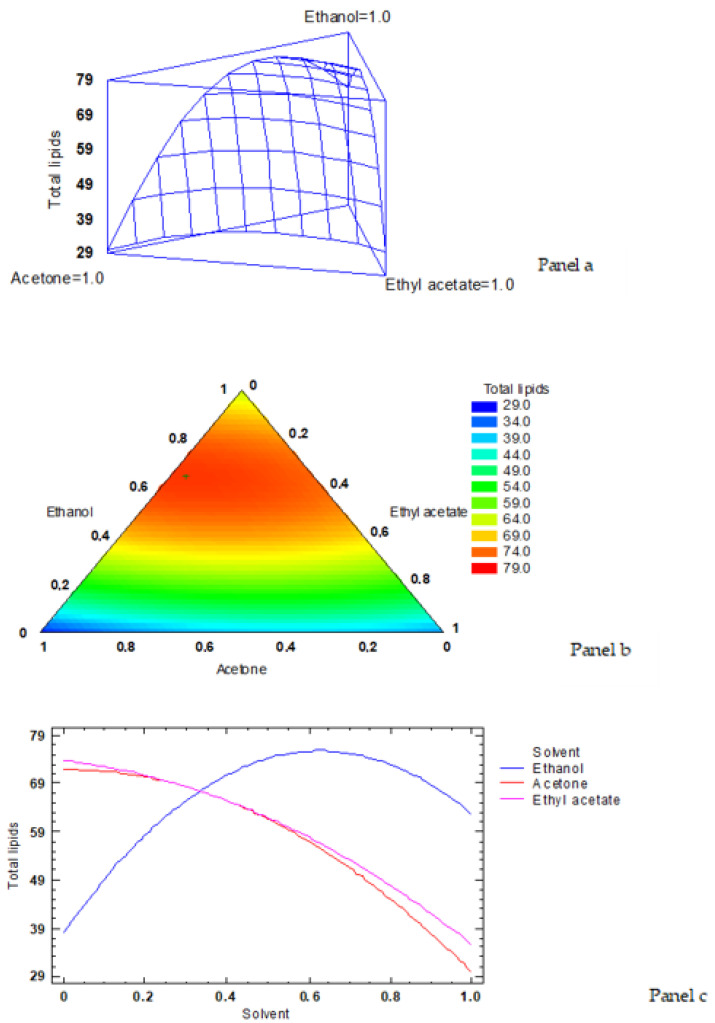
Application of the simplex-lattice design on the fitting model corresponding to lipid content (g·kg^−1^ by-products) results. **Panel a**: estimated response surface; **Panel b**: contours of estimated response surface; **Panel c**: trace plot for lipid content.

According to the simplex-lattice mixture design, the fitted model for lipid content led to the estimated response surface shown in Figure 1a. In it, the three experimental factors are the low-toxicity solvent ratios, and the height of the surface represents the lipid content value, the maximum value corresponding to 76.15 g·kg^−1^ by-products. An empirical coded equation could be used to model the effect of the low-toxicity solvent ratios. Thus, Equation (2) shows the fitted model that represents the estimated response surface of the total lipid content (g·kg^−1^ dry by-products) as a function of ethanol (E), acetone (A), and ethyl acetate (EA) ratios (0–1 range):

Lipid content = 62.6123 × E + 29.7123 × A + 35.5123 × EA + 109.9630 × E × A + 91.3631 × E x EA + 22.1631 × A × EA(2)

Figure 1b shows the contour response surface of the estimated response surface for the lipid value as a function of the low-toxicity solvent concentrations. The design is represented by a triangle where the three vertices represent the three pure solvents and the points placed on the triangle sides correspond to the binary mixtures. The solvent minimum level is 0 and the maximum is 1, corresponding to the pure solvent in coded variables. Each contour line represents the combinations of ethanol, acetone, and ethyl acetate ratios, which give a selected value for the lipid content. The combination of factor levels (i.e., solvent ratios) that maximised the response variable of the total lipid content over the indicated region was 0.642/0.318/0.040 (ethanol/acetone/ethyl acetate, respectively). As previously expressed, such a combination led to an optimised value of 76.15 g·kg^−1^ by-products (the cross in the red sector in Figure 1b indicates the maximum value of the lipid content), representing an 84% recovery of the yield corresponding to the traditional extraction procedure.

The trace plot for the lipid content is shown in Figure 1c. This plot shows the effect on the lipid yield of a ratio increase or decrease in any of the solvents employed. Taking into account the centroid point (i.e., 0.333 value for each solvent), the lipid content showed to increase as the ethanol ratio increased in the extracting system, while it decreased with increasing acetone and ethyl acetate ratios.

### 3.2. Tocopherol Compound Extraction

Independently of the extracting system applied, the analysis of the tocopherol fraction showed that the major component in the present squid by-products was α-tocopherol (Table 2). This result agrees with previous research on marine animals from natural diets [22]. Additionally, the presence of γ- and δ-tocopherol and the absence of β-tocopherol were also detected in all kinds of lipid extracts. Remarkably, the presence of β-, γ-, and δ-tocopherol has shown to be negligible in wild marine vertebrates and very low in invertebrates [22]. On the contrary, a remarkable presence of such three tocopherols has been detected in other kinds of food, such as those obtained from vegetable sources [45,46].

Values obtained for α-, γ-, and δ-tocopherol in the initial squid by-products were 1314.3 ± 67.3, 10.9 ± 0.5, and 3.8 ± 0.3 mg·kg^−1^ lipids, respectively. Values for α-tocopherol can be considered slightly higher than those reported for the by-products of the same squid species in a previous seasonal study (539.6–973.3 mg·kg^−1^ lipids) [30]. Additionally, current α-tocopherol levels were higher than those observed in different edible zones of megrim (9.8–14.1 mg·kg^−1^ lipids) (*Lepidorhombus whiffiagonis*) [47], as well as in the muscle tissues of wild marine species in general [22]. Contrary, α-tocopherol values obtained in the present study were substantially lower than those reported for wild blackspot seabream (*Pagellus bogaraveo*) (1,327–1,672 mg·kg^−1^ lipids) flesh, but higher than those observed in farmed individuals corresponding to this fish species (338–400 mg·kg^−1^ lipids) [42].

If data obtained for initial squid by-products are considered on a by-product basis, values for the three tocopherol molecules were 20.35 ± 1.04, 0.17 ± 0.01, and 0.06 ± 0.00 mg·kg^−1^ dry by-products, respectively. Lower levels than in the current work were detected for α-tocopherol in farmed (3.8–10.5 mg·kg^−1^ flesh) and wild (7–8.8 mg·kg^−1^ flesh) turbot (*Psetta maxima*) when studying different edible zones [48]. Lower levels of α-tocopherol (12.6–12.9 mg·kg^−1^ flesh) were also detected in farmed salmon (*Oncorhynchus mykiss*) muscle by Ortiz et al. [49]. On the contrary, levels observed for γ- (2.2–2.3 mg·kg^−1^ flesh) and δ-tocopherol (0.31–0.37 mg·kg^−1^ flesh) were higher than in the present study [49]. A previous study including a wide range of marine species revealed α-tocopherol values included in the 5.06–17.90 mg·kg^−1^ muscle range [19], which can be considered somewhat lower than the current α-tocopherol level.

All low-toxicity extracting systems led to higher (*p* < 0.05) values of α-tocopherol than in the case of traditional extraction when considering data obtained on a lipid basis (Table 2). This higher content can be explained on the basis that non-polar lipid classes (i.e., triacylglycerols and waxes) are not extracted entirely by the current low-toxicity solvents, leading to a relatively higher presence in the lipid extract of other lipid molecules, such as α-tocopherol. Comparisons among the different low-toxicity extracting systems tested showed the highest content (*p* < 0.05) in the lipid extract obtained with only acetone, followed by those lipid fractions obtained by employing ethyl acetate and acetone/ethyl acetate. On the contrary, the lowest (*p* < 0.05) α-tocopherol concentrations were detected in lipid extracts corresponding to ethanol-including systems.

As for α-tocopherol, all low-toxicity extracting systems led to higher (*p* < 0.05) γ-tocopherol recovery yields than the traditional procedure when taking into account the results obtained on a lipid basis (Table 2). Although contents were substantially lower than those obtained for α-tocopherol, a similar yield distribution among the solvent systems tested could be observed for γ-tocopherol. Thus, the highest average value was observed by employing acetone alone as an extracting system. High yields were also detected using ethyl acetate and acetone/ethyl acetate. As for α-tocopherol yields, the lowest values (*p* < 0.05) of γ-tocopherol were obtained by employing ethanol-including systems (i.e., ethanol, ethanol/acetone, ethanol/ethyl acetate, and ethanol/acetone/ethyl acetate).

The values obtained for δ-tocopherol were very low (Table 2). Thus, differences (*p* > 0.05) among extracting systems, i.e., traditional and all low-toxicity systems, could not be detected. However, and according to the results obtained for α- and γ-tocopherol, the highest average values were obtained using acetone alone as an extracting system (2.8 mg·kg^−1^ lipids). This average value was found to be higher than the one obtained using the traditional procedure. On the contrary, the lowest average values for low-toxicity solvents were detected in lipid extracts corresponding to ethanol-including systems (i.e., 2.0–2.1 mg·kg^−1^ lipids).

Table 2 also shows the contents of the different tocopherol compounds expressed on a by-product basis. In the case of α-tocopherol, low-toxicity systems led to valuable recovery values (88.0–97.7%) when compared to the lipid yield obtained with the traditional procedure; ethanol/ethyl acetate and acetone systems led to the highest and lowest average values, respectively. Concerning γ-tocopherol, high recovery values were also detected by the employment of the low-toxicity systems, which were included in the 80.0–95.0% range when compared to the traditional procedure. In this case, acetone/ethyl acetate and ethyl acetate alone led to the highest average values. Recovery values of δ-tocopherol were lower than in the case of α- and γ- compounds. Thus, yields included in the 25–75% range were detected when compared to the recovery values obtained by the traditional procedure. The ethanol/acetone system led to the highest average value.

When compared to the values obtained with the traditional procedure, low-toxicity solvent extraction led to different yields depending on the consideration of the lipid basis or the dry biomass basis. Such differences can be explained as a result of the ability of the different low-toxicity solvents to extract the different kinds of lipid molecules included in the squid by-products. As expressed above, data on a lipid basis would be influenced by the lower ability of the low-toxicity solvents tested for extracting non-polar lipid classes (i.e., triacylglycerols and waxes); thus, the presence in the lipid extract of relatively polar compounds, such as tocopherol compounds, would be higher than in the lipid extract obtained by the traditional procedure. In the case of considering data on a by-product basis, it is worth pointing out that yield values were included in a narrower range (13.8–15.5 and 0.16–0.20 mg·kg^−1^ dry by-products for α- and γ-tocopherol, respectively) than in the case of taking into account the lipid basis.

An important effect of low-toxicity solvent polarity was also detected in previous related studies. Gigliotti et al. [16] detected an increasing cholesterol presence by increasing the acetone ratio in an acetone/ethanol extracting system when applied to Antarctic krill (*E. superba*). In previous research [31], the lower polarity of acetone and ethyl acetate than the one of ethanol led to a higher ability to extract lipid classes, such as free fatty acids, free sterols, and triacylglycerols. A higher extractability in acetone of such lipid classes was explained on the basis that this solvent was the less polar of the three low-toxicity solvents tested in such study. Contrary to the present results, Li et al. [18] obtained a higher α-tocopherol recovery from microalga *S. dimorphus* by employing ethanol-containing systems (aq. 95% ethanol; ethanol/hexane, 3:2, *v*/*v*) than in the case of using ethyl acetate/hexane (1:1, *v*/*v*), methanol/hexane (1:0.8, *v*/*v*), or hexane alone.

Based on the simplex-lattice design developed, the variance analysis of data corresponding to α-tocopherol was carried out. As a result, the quadratic model showed to be statistically significant (*p* < 0.05) (Table 3). The variance analysis also indicated that the fitted model explained 98.73% of the variability of the α-tocopherol content (R-squared value), the adjusted R-squared value being 97.93%. Furthermore, the SEE showed that the standard deviation of the residuals was 0.0904. Based on such am SEE value, it could be assumed that the forecasts and predictions were accurate.

**Figure 2:**
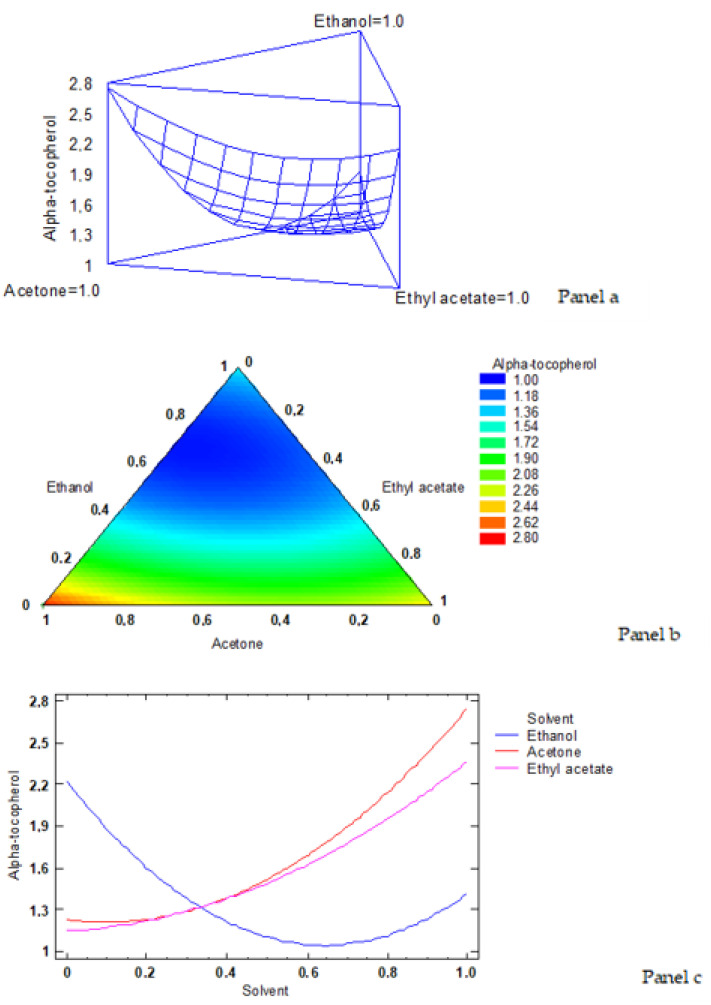
Application of the simplex-lattice design on the fitting model corresponding to the α-tocopherol content (g·kg^−1^ lipids). **Panel a**: estimated response surface; **Panel b**: contours of estimated response surface; **Panel c**: trace plot for α-tocopherol content.

According to the simplex-latex mixture design, the fitted model for α-tocopherol led to the estimated response surface shown in Figure 2a. In it, the three experimental factors were the low-toxicity solvent ratios, and the height of the surface represented the α-tocopherol content, the maximum value of α-tocopherol corresponding to 2.75 g·kg^−1^ lipids. An empirical coded equation could be used to model the effect of the low-toxicity solvent ratios. Thus, Equation (3) shows the fitted model that represents the estimated response surface of the α-tocopherol content (g·kg^−1^ lipids) as a function of the ethanol (E), acetone (A), and ethyl acetate (EA) ratios (0–1 range):

α-tocopherol content = 1.4134 × E + 2.7484 × A + 2.3684 × EA−3.7378 × E × A−2.6578 × E × EA−1.3478 × A × EA(3)

Figure 2b shows the contours of the estimated response surface for α-tocopherol content as a function of the low-toxicity solvent concentrations. Each contour line represents the combinations of ethanol, acetone, and ethyl acetate, which gives a selected value for α-tocopherol content. Based on the optimisation of the independent variables, the combination of factor levels (solvent ratios) which maximised the response variable of the α-tocopherol content over the indicated region was 0.0/1.0/0.0 (ethanol/acetone/ethyl acetate, respectively). As previously expressed, such a combination led to an optimised value of 2.75 g·kg^−1^ lipids (the cross in the red area in Figure 2b indicates the maximum value of the α-tocopherol content).

The trace plot for the α-tocopherol content is shown in Figure 2c. This plot shows the effect on α-tocopherol values of a ratio increase in any of the low-toxicity solvents tested. Considering the centroid point (i.e., 0.333 value for each solvent), the α-tocopherol value increased as the acetone and ethyl acetate ratios increased in the extracting system, while it decreased by increasing the ethanol ratio.

The simplex-lattice design was also applied to data related to γ-tocopherol. Thus, the quadratic model showed to be statistically significant (*p* < 0.05) (Table 3). The variance analysis also indicated that the fitted model explained 99.05% of the variability of the γ-tocopherol content (R-squared value), the adjusted R-squared value being 98.46%. Furthermore, the SEE showed that the standard deviation of the residuals was 1.1773. On the basis of such an SEE value, it could be assumed that the forecasts and predictions were accurate.

**Figure 3:**
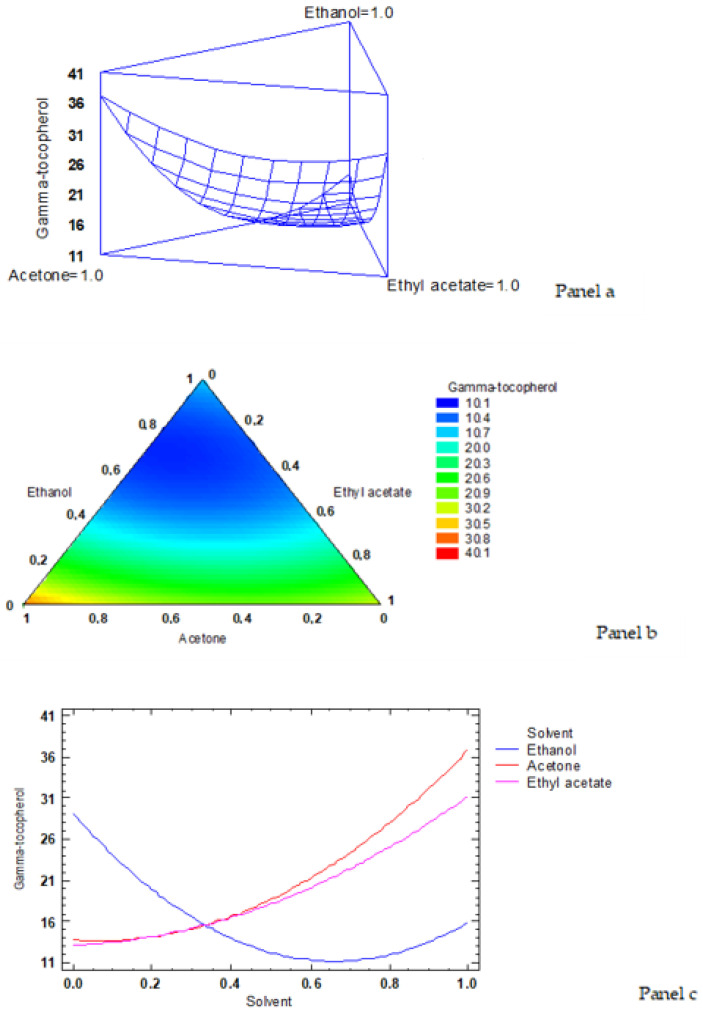
Application of the simplex-lattice design on the fitting model corresponding to the γ-tocopherol content (mg·kg^−1^ lipids). **Panel a**: estimated response surface; **Panel b**: contours of estimated response surface; **Panel c**: trace plot for γ-tocopherol content.

According to the simplex-latex mixture design, the fitted model for γ-tocopherol content led to the estimated response surface shown in Figure 3a. In it, the three experimental factors are the solvent ratios, and the height of the surface represents the γ-tocopherol value, the maximum value of γ-tocopherol content corresponding to 36.9 mg·kg^−1^ lipids. An empirical coded equation could be used to model the effect of the low-toxicity solvent ratios. Thus, Equation (4) shows the fitted model that represents the estimated response surface of the γ-tocopherol content (mg·kg^−1^ lipids) as a function of ethanol (E), acetone (A), and ethyl acetate (EA) ratios (0–1- range):

γ-tocopherol content = 15.9071 × E + 36.9071 × A + 31.2571 × EA − 52.9493 × E × A − 39.0493 × E × EA − 20.0493 × A × EA(4)

Figure 3b shows the contours of the estimated response surface for the γ-tocopherol content as a function of the low-toxicity solvent ratios. Each contour line represents the combinations of ethanol, acetone, and ethyl acetate which give a selected value for the γ-tocopherol content. Based on the optimisation of the independent variables, the combination of factor levels (solvent ratios) which maximised the response variable of the γ-tocopherol content over the indicated region was 0.0/1.0/0.0 (ethanol/acetone/ethyl acetate, respectively), with an optimised value of 36.9 mg·kg^−1^ lipids (the cross in the red area in Figure 3b indicates the maximum value of the γ -tocopherol content).

The trace plot for the γ-tocopherol content is shown in Figure 3c. This plot shows the effect on γ-tocopherol values of a ratio increase or decrease in any of the low-toxicity solvents employed. Considering the centroid point (i.e., 0.333 value for each solvent), the γ-tocopherol value increased as the acetone and ethyl acetate ratios increased in the extracting system, while it decreased by increasing the ethanol ratio.

The simplex-lattice design was also applied to data related to δ-tocopherol. Thus, the quadratic model showed to be statistically significant (*p* < 0.05) (Table 3). The variance analysis also indicated that the fitted model explained 80.55% of the variability of δ-tocopherol content (R-squared value), the adjusted R-squared value being 68.40%. Furthermore, the SEE showed that the standard deviation of the residuals was 0.1623. On the basis of such an SEE value, it could be assumed that the forecasts and predictions were accurate.

**Figure 4:**
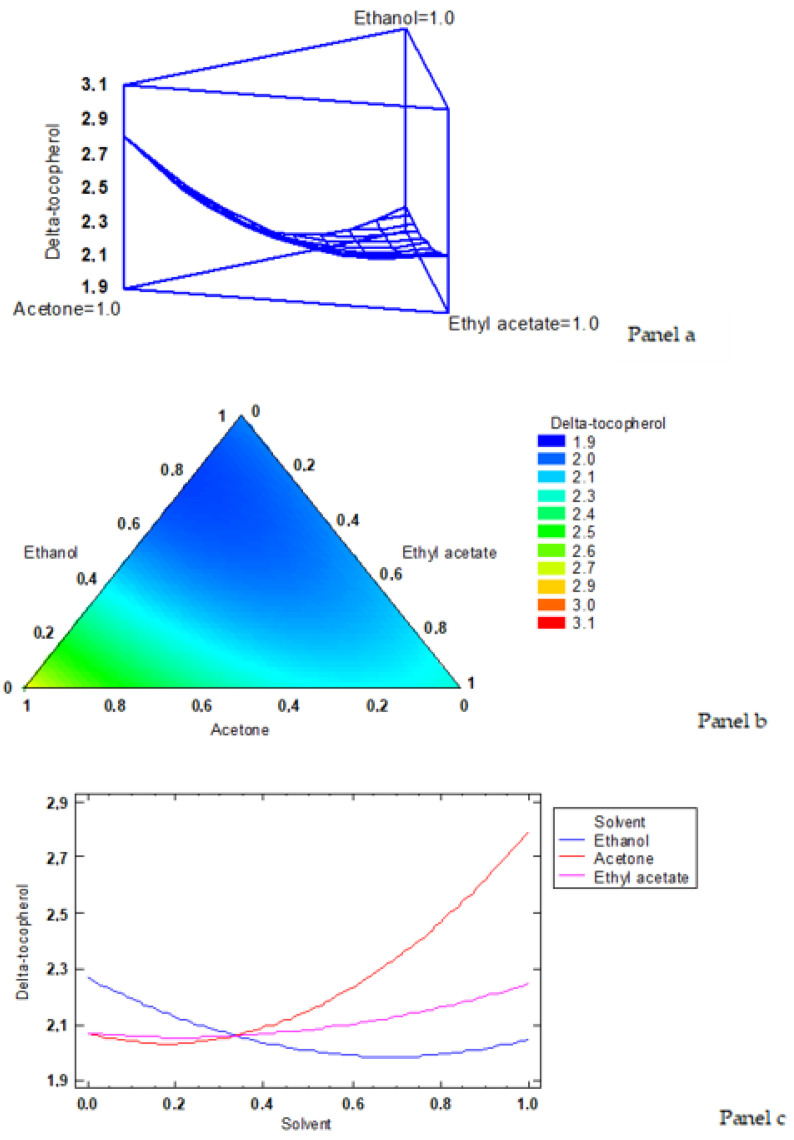
Application of the simplex-lattice design on the fitting model corresponding to the δ-tocopherol content (mg·kg^−1^ lipids). **Panel a**: estimated response surface; **Panel b**: contours of estimated response surface; **Panel c**: trace plot for δ-tocopherol content.

According to the simplex-latex mixture design, the fitted model for δ-tocopherol led to the estimated response surface shown in Figure 4a. In it, the three experimental factors are the low-toxicity solvent ratios, and the height of the surface represents the δ-tocopherol value, the maximum value of δ-tocopherol content corresponding to 2.79 mg·kg^−1^ lipids. An empirical coded equation could be used to model the effect of the low-toxicity solvent concentrations. Thus, Equation (5) shows the fitted model that represents the estimated response surface of the δ-tocopherol content (mg·kg^−1^ lipids) as a function of ethanol (E), acetone (A), and ethyl acetate (EA) ratios (0–1 range):

δ-tocopherol content = 2.04535 × E + 2.79535 × A + 2.24535 × EA − 1.40686 × E × A − 0.306858 × E × EA − 1.00686 × A × EA(5)

Figure 4b shows the contours of the estimated response surface for the δ-tocopherol content as a function of the low-toxicity solvent concentrations. Each contour line represents combinations of ethanol, acetone and ethyl acetate which give a selected value for the δ-tocopherol content. Based on the optimisation of the independent variables, the combination of factor levels (solvent ratios) which maximised the response variable of the δ-tocopherol content over the indicated region was 0.0/1.0/0.0 (ethanol/acetone/ethyl acetate, respectively), with an optimised value of 2.79 mg·kg^−1^ lipids (the cross in the yellow area in Figure 4b indicates the maximum value of the δ -tocopherol content).

The trace plot for the δ-tocopherol content is shown in Figure 4c. This plot shows the effect on δ-tocopherol values of a ratio increase or decrease in any of the ratios employed in the different low-toxicity solvents. Taking into account the centroid point (i.e., 0.333 value for each solvent), the δ-tocopherol value increased as the acetone ratio increased in the extracting system, while it decreased with increasing ethanol and ethyl acetate ratios.

## 4. Conclusions

The optimised lipid extraction was obtained by employing the 0.642/0.318/0.040 (ethanol/acetone/ethyl acetate) solvent ratio, respectively, leading to an 84% recovery of the total lipids extracted by the traditional procedure. In all extracting systems tested, the presence of α-, γ-, and δ-tocopherol compounds was detected, α-tocopherol being the most abundant. For α-, γ-, and δ-tocopherol compounds, the optimisation process showed that acetone extraction led to the highest concentrations in the lipid extract obtained (2736.5, 36.8, and 2.8 mg·kg^−1^ lipids, respectively). Taking into account the recovery yield on a by-product basis, values obtained for the three tocopherol compounds were included in the 88.0–97.7%, 80.0–95.0%, and 25–75% ranges, respectively, when compared to the traditional extraction.

Results obtained in the current study constitute a novel and promising basis to employ whole by-products from Patagonian squid as a source of total marine lipids and α-tocopherol compound, a reported chain-breaking antioxidant. To the best of our knowledge, no previous available research has addressed how to obtain this natural antioxidant compound from seafood by-products. The suitability of low-toxicity solvent systems tested was observed, matching present international interests in the search for alternatives for bioactive extracting methods that may reduce risks of chemical exposure to humans and the environment. Before subsequent use of by-products from the current squid species is to be carried out, international requirements concerning safety aspects (content on heavy metals, pesticides, and aromatic hydrocarbons) ought to be addressed.

The employment of an optimisation design has shown its usefulness in the present study, so that such advanced mathematical analyses can represent a useful tool during seafood extraction and processing in general in order to simplify the experimental conditions and foresee the optimised results to be obtained. Based on the valuable relevance of the current results, further studies including mathematical modelling are envisaged to optimise and scale-up the extraction conditions of total lipids and α-tocopherol from the present squid by-products.

## Figures and Tables

**Table 1:** Description of the different low-toxicity solvent systems applied for lipid extraction of lyophilised squid by-products according to the simplex-lattice mixture design.

Extracting Condition Number	Low-Toxicity Solvent Relative Content (0–1 Range)		
	Ethanol	Acetone	Ethyl Acetate
1	1	0	0
2	1	0	0
3	0.5	0.5	0
4	0.5	0.5	0
5	0.5	0	0.5
6	0.5	0	0.5
7	0	1	0
8	0	1	0
9	0	0.5	0.5
10	0	0.5	0.5
11	0	0	1
12	0	0	1
13	0.333	0.333	0.333
14	0.333	0.333	0.333

**Table 2:** Values * obtained for total lipid, α-tocopherol, γ-tocopherol, and δ-tocopherol in squid by-products by employing different lipid-extracting systems.

Extracting System	Total Lipid (g·kg^−1^ Dry By-Products)	α-Tocopherol (mg·kg^−1^ Lipids)	γ-Tocopherol (mg·kg^−1^ Lipids)	δ-Tocopherol (mg·kg^−1^ Lipids)
Chloroform/Methanol	90.6 ± 3.8 ^e^	1025.9 ± 9.9 ^a^ (15.52)	10.6 ± 0.1 ^a^ (0.20)	2.4 ± 0.6 ^a^ (0.04)
Ethanol	62.8 ± 2.9 ^c^	1399.9 ± 88.9 ^c^ (14.65)	15.8 ± 1.1 ^c^ (0.16)	2.0 ± 0.1 ^a^ (0.02)
Ethanol/Acetone	72.9 ± 3.0 ^d^	1199.3 ± 17.7 ^b^ (14.58)	13.8 ± 0.4 ^b^ (0.16)	2.1 ± 0.1 ^a^ (0.03)
Ethanol/Ethyl acetate	71.1 ± 0.5 ^d^	1278.0 ± 45.8 ^c^ (15.17)	14.4 ± 1.1 ^bc^ (0.17)	2.1 ± 0.0 ^a^ (0.02)
Acetone	29.9 ± 0.9 ^a^	2736.5 ± 67.2 ^e^ (13.66)	36.8 ± 0.8 ^e^ (0.18)	2.8 ±0.5 ^a^ (0.01)
Acetone/Ethyl acetate	37.4 ± 0.7 ^b^	2274.1 ± 10.8 ^d^ (14.20)	29.7 ± 0.3 ^d^ (0.19)	2.2 ± 0.1 ^a^ (0.01)
Ethyl acetate	35.7 ± 1.1 ^b^	2357.4 ± 60.0 ^d^ (14.05)	31.1 ± 1.2 ^de^ (0.19)	2.3 ± 0.0 ^a^ (0.01)
Ethanol/Acetone/Ethyl acetate	69.0 ± 1.5 ^d^	1196.0 ± 16.5 ^b^ (13.78)	14.2 ± 0.4 ^bc^ (0.16)	2.1 ± 0.0 ^a^ (0.02)

* In each column, average values (*n* = 4) ± standard deviations followed by different lowercase letters denote significant differences (*p* < 0.05). For tocopherol compounds, values in brackets correspond to average values expressed as mg·kg^−1^ dry by-products.

**Table 3:** ANOVA application of the simplex-lattice mixture design of fitting model to response variables (total lipid values and contents on α-, γ-, and δ-tocopherol).

Quadratic Model	Response Variables			
	Total Lipid	α-Tocopherol	γ-Tocopherol	δ-Tocopherol
Model d.f.	6	6	6	6
*p*-value	0.0000	0.0000	0.0000	0.0000
Error d.f.	8	8	8	8
Standard error of the estimate	2.0072	0.0904	1.1773	0.1623
R-squared (%)	99.25	98.73	99.05	80.55
Adjusted R-squared (%)	98.79	97.93	98.46	68.40

## Data Availability

Data is contained within the article.
